# Science of malaria elimination: using knowledge of bottlenecks and enablers from the Malaria Elimination Demonstration Project in Central India for eliminating malaria in the Asia Pacific region

**DOI:** 10.3389/fpubh.2023.1303095

**Published:** 2024-01-18

**Authors:** Harsh Rajvanshi, Mrigendra P. Singh, Praveen K. Bharti, Ram Shankar Sahu, Himanshu Jayswar, Pallavi Jain Govil, Anup Anvikar, Xavier Xuanhao Chan, Amita Chebbi, Sarthak Das, Altaf A. Lal

**Affiliations:** ^1^Asia Pacific Leaders' Malaria Alliance, Singapore, Singapore; ^2^Foundation for Disease Elimination and Control of India, Mumbai, Maharashtra, India; ^3^Indian Council of Medical Research – National Institute of Malaria Research, New Delhi, India; ^4^District Malaria Office, Mandla, Madhya Pradesh, India; ^5^Directorate of Health Services, Government of Madhya Pradesh, Bhopal, India; ^6^Department of Tribal Welfare, Government of Madhya Pradesh, Bhopal, India

**Keywords:** Tribal Malaria, malaria elimination, MEDP, Mandla, public-private partnership

## Abstract

Malaria poses a major public health challenge in the Asia Pacific. Malaria Elimination Demonstration Project was conducted as a public-private partnership initiative in Mandla between State government, ICMR, and FDEC India. The project employed controls for efficient operational and management decisions. IEC campaigns found crucial in schools and communities. Capacity building of local workers emphasized for better diagnosis and treatment. SOCH mobile app launched for complete digitalization. Better supervision for Indoor Residual Sprays and optimized Long Lasting Insecticidal Nets distribution. Significant malaria cases reduction in Mandla. Insights from MEDP crucial for malaria elimination strategies in other endemic regions of the Asia Pacific.

## Introduction

Malaria, caused by *Plasmodium* parasites and transmitted by Anopheles mosquitoes, remains a significant health concern in the Asia Pacific region, particularly in India. The diverse climates in the region create ideal breeding grounds for these mosquitoes, especially in challenging terrains.

**Figure 1:**
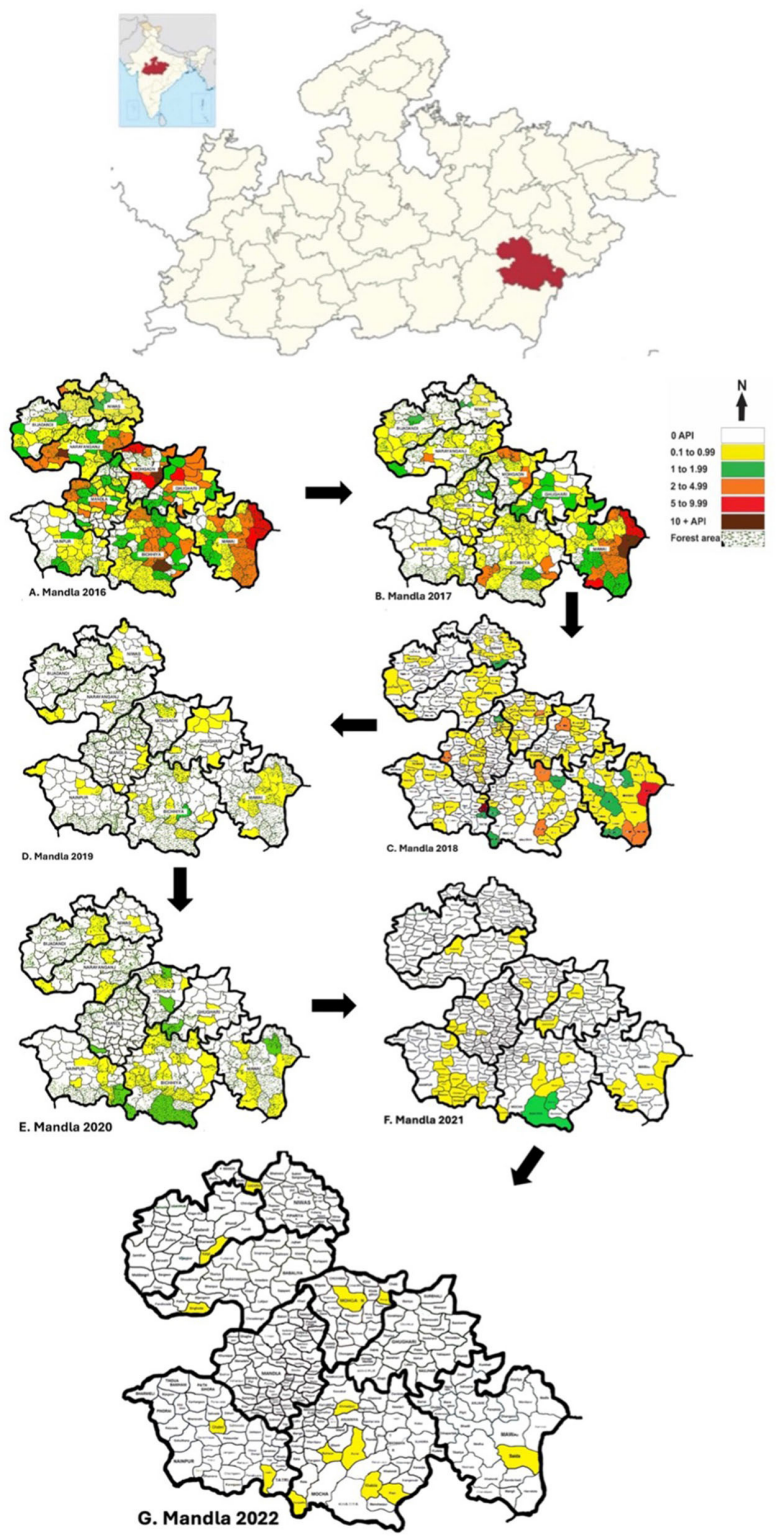
Map showing the location of the state of Madhya Pradesh in Central India. Madhya Pradesh is further expanded showing the location of Mandla district split into nine development blocks, each showing the transition of malaria endemicity from 2016 to 2022. Out of the total 297 sub-centers, 143 sub-centers were free of malaria in 2017, which increased to 198 in 2018, 211 in 2019, 243 in 2020, 262 in 2021, and 283 in 2022. The district had 8 hotspots for malaria (5+ API) in 2016, which were reduced to 3 in 2017, 2 in 2018, and 0 in 2019.

Mandla district in Madhya Pradesh, India, serves as a case study for malaria elimination efforts. Located in the Satpura mountain range with a significant tribal population, it faces perennial malaria transmission due to the Narmada River and its tributaries (1) (Figure 1).

Historically, Mandla was a high-prevalence malaria-endemic district grouped under category 3 districts by the Indian National Vector Borne Disease Control Programme. However, between 2015 and 2020, indigenous malaria cases decreased significantly from 3,901 to 127. This reduction is attributed to various interventions applicable to similar hard-to-reach areas with poor socioeconomic indicators and indigenous populations (2). The principal malaria vector species are *Anopheles culicifacies* (year-round presence) and *Anopheles fluviatilis* (seasonal presence) (3).

Scheduled Tribes constitute 8.6% of India's population, and about 70% of the country's malaria cases are reported in these tribal areas. This high prevalence is attributed to risk factors such as difficult terrain, dense forest cover, poor socio-economic conditions, and limited access to healthcare services (1, 4).

The National Center for Vector Borne Diseases Control (NCVBDC) implemented the Tribal Malaria Action Plan as part of its National Strategic Plan for Malaria Elimination 2017–2022. Furthermore, the Malaria Elimination Demonstration Project (MEDP) in Mandla district was initiated as a public-private partnership. The project aimed to demonstrate malaria elimination in the district and utilize the findings to inform malaria elimination strategies at state and national levels (5).

**Figure 2:**
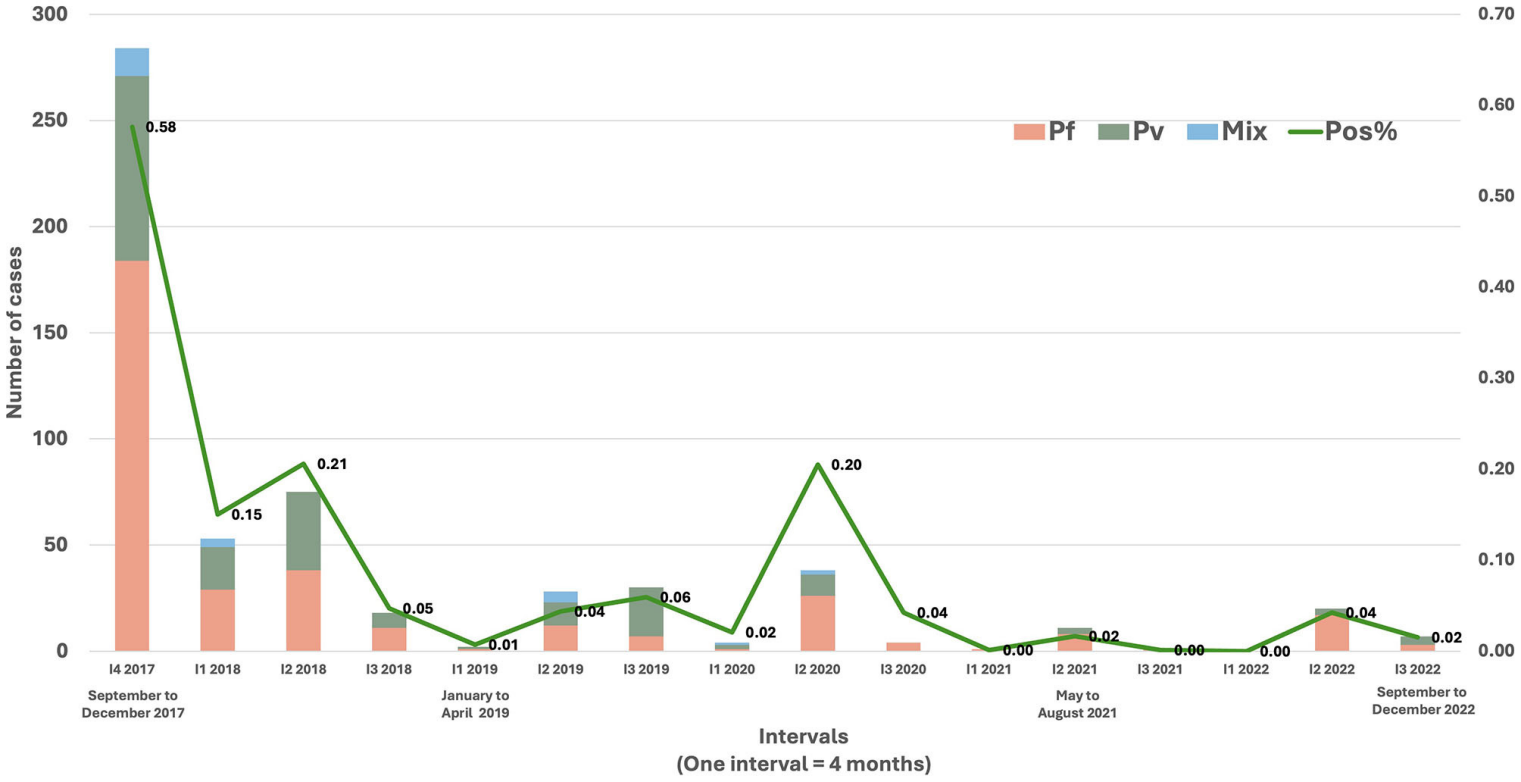
Trend of indigenous malaria cases in Mandla district from September 2017 to December 2022. The data is depicted in 4-month intervals, comprising three intervals in 1 year (e.g., September to December = One interval). MEDP ceased its operations in March 2021 and the malaria elimination activities post-March 2021 were driven by the District Malaria Office, Govt. of Madhya Pradesh.

Data from MEDP showed a 91% reduction in indigenous cases from September 2017 to March 2021. The project reported 18 months with no malaria out of 43 months of field operations in the district (Figure 2). This was attributed to surveillance, capacity building, vector control, and social and behavioral change communication, supplemented by robust operational and management controls (6–10).

This paper critically examines the progression of malaria cases in Mandla district to identify key bottlenecks and enablers in malaria elimination, which can be used for similar endemic areas within the Asia Pacific region. The knowledge of social, environmental, entomological, and epidemiological, as well as the implementation of case management and vector control strategies constitute the Science of Malaria Elimination, which is imperative for devising effective malaria elimination strategies.

## Technical bottlenecks and enablers

### Low-density malaria infections

#### Bottleneck

LDMI is defined as an infection where parasitaemia is missed by conventional diagnostic methods, such as microscopy and rapid diagnostic test (RDT), but identified by the more sensitive Polymerase Chain Reaction (PCR) diagnostic instrument (11). The presence of sub-microscopic or LDMI poses a challenge to eliminating malaria because individuals who harbor LDMI can continue to be the source of gametocytes for sustained malaria transmission. So far, studies using various molecular methods in various transmission settings have reported a prevalence of sub-microscopic *P. falciparum* ranging from 5 to 50% (12).

#### Enabler

MEDP integrated the Mass Screening and Treatment (MSaT) strategy in its surveillance and case management toolkit. In the absence of concrete national guidelines for MSaT, the project tested Cluster Combination Approaches (CCA) to generate evidence on the role of LDMIs in malaria transmission in Mandla district (13). The CCA screened the entire population (irrespective of malaria symptoms) within a defined geographical area with varying endemicities and hard-to-reach areas. Simultaneously thick and thin blood smears and blood spots were collected for further analysis using microscopy and species specific nested PCR for diagnosis of malaria parasites in a subset of the samples (11).

The MSaT revealed that there were significantly fewer asymptomatic malaria positive cases in areas with low malaria endemicity (API<1) as compared to other clusters of higher malaria endemicity and hard-to-reach areas. Additionally, the prevalence of malaria in febrile cases detected by PCR was 2.50% (436/17,405) vs. 1.13% (196/17,405) by RDT. A statistically significant (*p* < 0.001) increase in the number of LDMI cases was observed among subjects with a mean duration of fever of 3 days (11, 13).

### Classification of cases

#### Bottleneck

WHO's malaria elimination certification requires no indigenous transmission of malaria cases for 3 consecutive years (14), which has been achieved by countries like Sri Lanka (15) and China (16) in the Asia Pacific region. This makes the investigation and classification of malaria cases a necessity for the programs in the region. For example, Indonesia introduced sub-national malaria elimination certification (17), while India followed surveillance guidelines, limiting case investigation to certain districts (5). When Mandla transitioned to a Category 2 district, it faced challenges in classifying malaria cases without comprehensive guidelines, making it difficult to distinguish between indigenous and imported cases (6).

#### Enabler

MEDP developed comprehensive case classification guidelines in consultation with the National Vector Borne Disease Control Programme (NVBDCP) to determine the geographic nature of the infection. These guidelines were used by the District Malaria Office and MEDP staff from 2018 to 2021 (6). Of all the cases investigated, 20% of malaria cases were classified as imported/introduced.

### Social and behavioral change communication strategy

#### Bottleneck

The Mandla district followed SBCC outlined in the India NSP. It lacked the context-specific modifications for the culturally diverse district of Mandla, with various tribal sub-groups.

#### Enabler

In 2017, community members and MEDP field staff collaborated to develop and implement the IEC-based communication strategy “*for the people, by the people*”. The IEC campaigns utilized culturally-based communication instruments, such as integrated awareness cum treatment booths in weekly community markets; calendars, posters, and flipbooks with malaria prevention messaging; and job-aids for field workers. Lessons learned from previous studies conducted by ICMR-NIRTH in tribal areas were a major factor in selecting interpersonal communication as an appropriate IEC strategy for Mandla (18, 19).

### Treatment of *P. vivax* infections

#### Bottleneck

The present malaria elimination program in India and most Asia Pacific countries rely on low-dose Primaquine (15 mg/kg body weight) to treat the liver stage of the infection and to prevent the recurrence (20). The novel drug, Tafenoquine, which offers a single-day solution as a replacement to Primaquine, has been approved in some countries such as the US, Brazil, and Thailand (21) and is under various stages of assessment and approvals in the Asia Pacific region. The malaria-endemic countries in the region may have to rely on Primaquine till then, which due to its longer duration and higher pill load, poses a challenge toward ensuring treatment compliance.

#### Enabler

The MEDP adopted the existing approved and proven malaria drug policy in India, which included low-dose PQ. Anti-malaria drugs were administrated under direct observation by the MEDP field staff for both *P. falciparum* and *P. vivax* to ensure complete treatment compliance. The *P. vivax* prevalence averaged at 38% from 2017 to 2020 in Mandla (22). The mobile application Solutions for Community Healthworkers (SOCH) (23) and a dedicated supervisory staff assisted the field staff to achieve an almost perfect treatment compliance rate (6).

## Programmatic bottlenecks and enablers

### Monitoring frameworks

#### Bottleneck

The Lancet Commission on Malaria Eradication has strongly recommended the need for robust monitoring and accountability frameworks across all activities of the malaria elimination programs (24). Like most Asia Pacific countries, in India, the regular monitoring frameworks for malaria elimination are based on the NSP. However, inadequate tools, lack of context-specific modifications, and poorly implemented multi-level monitoring plans pose barriers to the successful implementation of the plans (5).

#### Enabler

MEDP worked with the District Malaria Office to create simple, lean, and robust monitoring tools in Mandla. Some of the examples include the 30-point monitoring checklist (25), spot-inspections and viva-voce (22), Advance Tour Plans (22), a digital mobile application-based surveillance, supply chain, and HR management system (23), weekly activity and monthly review reports (22), vector control monitoring checklists (26), and the annual reviews by the Malaria Elimination Advisory Group (22). These tools and techniques were developed, piloted, implemented, and the results were shared, published and disseminated at national/international meetings and peer-reviewed journals.

### Capacity building of frontline staff

#### Bottleneck

Community Health Workers in the Asia Pacific region have a primary responsibility for detecting and treating malaria cases (27). A needs-assessment study in Mandla found that only 15 and 10% of Accredited Social Health Activists (ASHAs) correctly identified RDTs with *P. vivax* and *P. falciparum* positive results, respectively. For *P. vivax* cases, only 38.2% of ASHAs administered PQ for 14 days, 19.1% lacked RDTs for diagnosis, and 47.7% lacked Artemisinin-based Combination Therapies (ACTs) for *P. falciparum* malaria treatment (28).

#### Enabler

After the 2017 needs assessment, MEDP and the District Malaria Office designed and implemented a training curriculum for ASHAs in Mandla. This resulted in an 80% improvement in ASHAs' ability to diagnose malaria using RDTs, among other achievements (29). The training curriculum was adapted from successful modules used for Village Malaria Workers in MEDP, where 94.3% of candidates qualified after a single training session (with a 70% passing mark). A “shadowing” technique was employed, with new recruits shadowing supervisors for a week before officially starting work (30). Additionally, “job-aids” were distributed to all ASHAs in Mandla, which were pocket-sized reference tools containing malaria-related diagnostic, treatment, and prevention guidelines (22).

### Surveillance and reporting systems

#### Bottleneck

Data reporting systems in countries can be paper-based or digital. As of 2017, Mandla district in India used a paper-based reporting system for malaria elimination activities. This system lacked real-time data sharing and posed risks of errors during data collation and digitization from paper forms (22).

#### Enabler

To address this challenge, MEDP developed the Solution for Community Healthworkers (SOCH) mobile app in Hindi and English. SOCH, a native Android app, ensured data quality and integrity through self-validation tools. Starting in August 2018, MEDP successfully digitized routine surveillance, attendance, tour plans, and supply chain management, achieving a 99.6% accuracy rate in mobile data collection. Real-time data monitoring was available on a desktop dashboard, with Key Performance Indicators readily accessible (23). SOCH is adaptable for various disease surveillance and control programs and languages, including English and other Indian or international languages. It also includes indicators for Indoor Residual Spraying (IRS), Long-Lasting Insecticide Nets (LLIN), and community-resident involvement in surveillance.

### Utilization of vector control tools

#### Bottleneck

A recent global assessment on the capacity of NMCPs to implement vector control interventions has revealed that only 8% of programs have sufficient capacity to implement vector surveillance, with only half of them able to implement LLINs and IRS (31). A baseline assessment for IRS done in Mandla in 2017 revealed several gaps toward the implementation of the spray (26) and marked the effectiveness of IRS (30-day mosquito knockdown rate) at 40.6% (3). The same assessment also revealed that only 34% of the LLINs were being used regularly, and less than half (41.6%) of recipients received a demonstration on the correct usage of LLINs (26).

#### Enabler

To improve the effectiveness of vector control interventions, the Govt. of MP requested “supportive supervision” from MEDP during IRS and LLIN distributions. This included supervision of preparedness for sprays, spraying techniques, demonstration of LLIN usage, post-distribution usage of LLINs etc. Indicators related to preparedness of spraying went by up to 70%, the methodology of spray preparation improved from 50 to 90%, and the spraying technique such as distance between wall and lens, spray direction, uniformity, overlapping of swath etc. increased from 54 to 80%. Post 8-month distribution of LLINs, improvement was seen in regular usage by 28%, a reduction in reported side-effects by 64% and an increase in awareness by 97% (26).

### Issues with alternate systems of medicine

#### Bottleneck

The reports of rampant issue of unlicensed practitioners dispensing questionable anti-malarial drugs is a major problem in the Asia Pacific (32, 33). In Mandla district in 2017, the majority of the population relied on unlicensed private practitioners and facilities. There were only seven registered allopathic practitioners, 46 registered AYUSH practitioners and 366 unqualified practitioners. Remarkably, the highest concentrations of unqualified practitioners were in malaria-burdened areas (22).

During 2017–18, an unapproved and untested homeopathic drug called *Malaria Officinalis 200* was introduced in malaria-endemic areas, including Mandla, through AYUSH dispensaries (34). This drug posed a threat to malaria elimination efforts, as the community preferred this “preventive” therapy over blood examinations and radical treatment.

#### Enabler

MEDP established a sentinel surveillance network in collaboration with private practitioners in Mandla district as part of its comprehensive surveillance strategy. These practitioners were enrolled, and data on outpatient cases, fever cases, and malaria positives were recorded. Between April 2019 and May 2020, 8,416 patients were tested for malaria, with only 11 testing positive (0.13%). The key strategy was to support private practitioners in testing and treating their patients without project scrutiny (6).

MEDP also advocated against the distribution of *Malaria Officinalis 200* to government policymakers, highlighting its unapproved status from the national program and Central Drugs Standard Control Organization. This advocacy successfully halted the drug's use, removing a potential barrier to malaria elimination efforts in Mandla district.

### Issues with procurement of drugs and diagnostics

#### Bottleneck

Supply chain management challenges are common in the region due to factors like disease endemicity, logistics costs, commodity expiry, and minimum order quantities. Local solutions offer potential solutions to these issues (35).

In Mandla district, the District Malaria Office and MEDP managed malaria elimination efforts through a public-private partnership. Procuring ACTs required a minimum order of 125,000 combi-packs, which became problematic as malaria cases decreased, and it was challenging to supply all 235 Village Malaria Workers with age-specific doses. Moreover, the use of lancets with triangular blades resulted in painful testing and potential infection risks, making community members reluctant to undergo malaria testing.

#### Enabler

The Madhya Pradesh government addressed both supply issues. They resolved the ACT supply problem by transferring stock from nearby districts to Mandla and leveraging the state's mass demand to meet minimum order quantity requirements. Regarding lancets, MEDP advocated for change based on community feedback and the state replaced them with high-quality lancets in subsequent supplies to Mandla district.

## Policy and advocacy-related bottlenecks and enablers

### Lack of year-round advocacy initiatives

#### Bottleneck

Mandla district observed “Anti Malaria Month (AMM)” in June each year as per the national guidelines (5). This involved 30 days of advocacy and behavior change communication activities throughout the district at various levels. However, no monitoring system was in place to determine the impact of AMM. Additionally, Mandla experienced a perennial transmission of malaria, which was not limited to specific months or periods.

#### Enabler

Instead of a month-long AMM, MEDP focused on year-round advocacy and Social and Behavior Change Communication (SBCC) activities throughout the district. These included SBCC/Testing and Treatment booths at the weekly community markets, SBCC initiatives focusing on middle-school children, and inter-personal communication with the community leaders and residents during fortnightly active surveillance (door-to-door visits). In a study done in Mandla to assess the MEDP communication strategy, it was concluded that the combination of “Pull” by the community and “Push” by the MEDP field workers was instrumental in improving malaria elimination outcomes in a timely manner (19). Additionally, MEDP organized in-person dissemination of monthly reports with the highest government officials in the district, such as the District Magistrate, Superintendent of Police, and Chief Medical and Health Officer to keep malaria elimination high on the agenda of the district administration.

### Inadequate inter-sectoral coordination

#### Bottleneck

In the Asia Pacific, the vertical nature of malaria control programs has contributed to their success, but the last mile of elimination requires efforts that break down this verticality and promote inter-sectoral coordination and cooperation (36).

For example, in Kanha National Park in Mandla and Balaghat districts (both highly endemic), surrounding villages faced a high risk of forest malaria transmission. The surveillance network did not cover park staff, lacked coordination with the forest department (22), and exhibited inadequate inter-sectoral coordination with Auxiliary Nurse Midwives (ANMs) responsible for maternal and child health activities and active door-to-door surveillance.

#### Enabler

MEDP organized special MSaT campaigns in collaboration with the Forest Department and District Malaria Office, diagnosing and treating asymptomatic malaria among people living in the national park. Although regular surveillance was not feasible in restricted park zones, effective coordination with district authorities expanded the surveillance network. MEDP integrated the malaria elimination mandate with government assignments like pulse-polio rounds and COVID-19 surveillance (6, 7).

To ensure sustainable and replicable interventions, MEDP resolved bottlenecks creatively. They published their operational study design (22) and malaria elimination model (37), utilizing existing state government systems and strategies from the National Framework for Malaria Elimination (38) and the National Strategic Plan (5) with minimal additional costs. After the project's completion, the state government adopted MEDP's best practices in other districts, yielding positive results.

## Conclusion

The Malaria Elimination Demonstration Project (MEDP) in India's Mandla district achieved near elimination of indigenous malaria transmission, successfully identifying and managing imported cases using current tools. The project did not ascertain the impact of its vector-control strategies on other diseases like Dengue and Chikungunya. It remains possible that effective malaria-focused vector control may benefit control of other diseases. Given Mandla's similarities with various Asia Pacific regions, the insights from this project are deemed valuable for regional malaria elimination. Essential components include a trained workforce, comprehensive case guidelines, culturally apt communication strategies, private sector inclusion, enhanced monitoring frameworks, and digital tools like SOCH. Supported interventions included enhanced Indoor Residual Spraying and Long Lasting Insecticidal Nets. With these strategies, several peer-reviewed publications were produced and made accessible for public utility. MEDP's success underscores the role of integrated approaches, technology, community involvement, and public-private collaborations in tackling malaria and other diseases in the region.

## Data availability statement

The original contributions presented in the study are included in the article/supplementary material, further inquiries can be directed to the corresponding authors.

## Author contributions

HR: Conceptualization, Writing – original draft. MS: Data curation, Software, Visualization, Writing – review & editing. PB: Writing – review & editing. RS: Writing – review & editing. HJ: Writing – review & editing. PG: Supervision, Writing – review & editing. AA: Supervision, Writing – review & editing. XC: Supervision, Writing – review & editing. AC: Conceptualization, Supervision, Writing – review & editing. SD: Supervision, Writing – review & editing. AL: Conceptualization, Investigation, Project administration, Supervision, Writing – review & editing.
